# The validation of a French-language version of the Aging Perceptions Questionnaire (APQ) and its extension to a population aged 55 and over

**DOI:** 10.1186/1471-2318-12-17

**Published:** 2012-04-30

**Authors:** Isabelle Ingrand, Jean Luc Houeto, Roger Gil, Hannah Mc Gee, Pierre Ingrand, Marc Paccalin

**Affiliations:** 1 Pôle Biologie, Pharmacie et Santé Publique, Centre Hospitalier Universitaire de Poitiers; Université de Poitiers, Poitiers, France; 2 INSERM, CIC-P 802, Centre Hospitalier Universitaire de Poitiers, Poitiers, France; 3 Service Neurologie, Centre Hospitalier Universitaire de Poitiers Université de Poitiers, Poitiers, France; 4 Centre Mémoire de Ressources et de Recherche, Centre Hospitalier Universitaire de Poitiers; Université de Poitiers, Poitiers, France; 5 INSERM U 1084, Laboratoire Neurosciences Expérimentales et Cliniques; Université de Poitiers, Poitiers, France; 6 Department of Psychology, Division of Population Health Sciences, Royal College of Surgeons in Ireland, Dublin, Ireland; 7 Service de Gériatrie, Centre Hospitalier Universitaire de Poitiers; Université de Poitiers, Poitiers, France; 8 EA3808, Université de Poitiers, Poitiers, France

## Abstract

**Background:**

Several studies have shown the influence of the perceptions of aging on the cognitive functioning and the mental and physical health of older people. These relationships have not to date been studied in France where validated instruments are lacking. The primary objective of this study was to validate a French-language version of the Aging Perceptions Questionnaire (APQ) in the French general population aged 65 and over. The secondary objective was to study the stability of the dimensions of this instrument among participants aged 55 to 64.

**Methods:**

The study was proposed to the cohort of the *Observatoire Régional du Vieillissement* (OPREVI) (observatory of aging), located in a small town in Poitou-Charentes (western France). An anonymous questionnaire including the APQ was sent by mail to inhabitants aged 55 and over. The original English language APQ was described with adults aged 65 and older. It has 32 items distributed on 7 dimensions: *timeline chronic* and *cyclical*, *positive* and *negative consequences*, *positive* and *negative control* and e*motional representations.*

**Results:**

656 adults participated in this survey (286 men, 370 women). Among those aged 65 and over (n = 394), the seven-factor structure estimated by confirmatory factor analysis was coherent with original findings. Internal consistency as evaluated by Cronbach alpha, was between 0.83 for *consequences negative* and 0.52 for *control negative.* Several dimensions were strongly correlated. Among participants aged 55 to 64 (n = 262), the same factorial model yielded an acceptable fit. Multi-group confirmatory factor analysis concluded to approximate factorial invariance between the two age groups with a null delta in comparative fit index.

**Conclusion:**

This study among French people aged 65 and over, added further evidence of the multidimensional structure of the French version of the APQ which is superimposed to the dimensions of the original Irish version. The same factorial structure applies acceptably to the younger group (aged 55–64). The OPREVI study is ongoing, and will collect data on the physical, material and social characteristics of participants. It will therefore be possible to analyse the variables associated with the perceptions of aging. On the basis of an individual's perceptions of aging as captured by this questionnaire, and his or her clinical profile, tailored multi-dimensional assistance could be made available aiming to provide incentives to anticipate or to adapt to difficulties.

## Background

The systematic association of aging with deterioration does not contribute to an optimistic perception of growing old. Several longitudinal studies have shown the influence of perceptions of aging on cognitive functioning, and the mental and physical health of older people [1-4]. An optimistic view of aging and a better self-evaluation are associated with lower mortality [2,5]. In addition, individuals with a positive perception of growing old more readily adopt appropriate coping strategies [6-8].

The relationships between perceptions of aging and consequences on physical or mental health and on health-related behaviours have not to date been studied in France. Each society disseminates a specific image of aging which can affect individual viewpoints. It is unclear whether age perception has the same value in all European countries. Differences between cultures can be expected, as collectivist or individualistic culture or individual responsibility for health and social welfare. The individual adaptation strategies have to be studied in the cultural context and it is difficult to export theories from one cultural context to another [9]. Knowledge concerning these phenomena could help to establish interventional strategies to optimise the way in which people cope with growing old. The APQ (Aging Perceptions Questionnaire) is a recently developed multi-dimensional scale [10] with 7 dimensions which explores the respondent's views about aging on the basis of Leventhal's self-regulation model [11]. This model helps to examine the self-regulation of experience of health through a series of dimensions. Still this concept has only been applied in the context of health threat. As aging is not a health threat but a normal stage, it appeared useful to study self-adaptation in the context of aging.

Our interest was also in perceptions of aging among younger adults <65. We expected that their perception of aging could be influenced by the perspective of retirement. As we planned to study the successful aging from age 55, we needed a validation starting at 55. We think it too late to consider successful aging at age 65 if we want to plan interventions and try to modify self-perceptions of aging. This 55 and over age group was also taken into consideration because of the development of the 2007–2009 *"Bien Vieillir"* (“growing older better”) program in France, which focuses particularly on those aged 55 to 70 [12]. Middle-aged persons should be the main target of preventing strategies aimed to prepare and anticipate the natural effects of aging.

The main objective of this study was thus to validate a French-language version of the APQ in the French general population aged 65 and over as a first step towards research on the determinants of successful aging. The secondary objective was to study the stability of the dimensions of this instrument among those aged 55 to 64.

## Methods

### Study population and implementation

The French-language questionnaire was proposed to the cohort of the *Observatoire Régional du Vieillissement en Poitou-Charentes* (OPREVI) (observatory of aging), which aims to study the aging of a population aged 55 and over, through the demographic, social, psychological and medical determinants of the quality of life [4]. This study, based in the small town of Neuville-de-Poitou (4892 inhabitants) in the Poitou-Charentes region in western France, was initiated in 2008. In the first stage the observatory and its objectives, to analyse the links between demographic, social, psychological and medical variables and the quality of life [4], were presented to the medical and social personnel in the area, and then to the population in public meetings that took place from December 2008 to May 2010. In May 2010 a letter detailing the study was sent to all inhabitants identified from the electoral register, aged 55 or older (1506 individuals) accompanied by a consent form for participation, or to signal the desire not to participate. Then an anonymous questionnaire was sent to all the respondents by mail. The APQ was included in this first dispatch. The socio-demographic characteristics collected were age, marital status and level of education. In case of agreement to participate but difficulty in reading or writing responses, a third party could assist. A telephone number was available to answer questions and provide any information required. In case of non-response, a telephone reminder was given.

The study was approved by the French regulatory authorities, the *Comité Consultatif sur le Traitement de l'Information en matière de Recherche dans le domaine de la Santé* (CCTIRS) (consultative committee on information processing in health research) in July 2009, and the *Commission Nationale Informatique et Libertés* (CNIL) (commission for protection of private data) in December 2009.

### The Aging Perceptions Questionnaire (APQ)

We used a French-language version of the questionnaire published by McGee's team [10]. The APQ was translated by a bilingual native English speaker specialised in the translation of subjective measures, with detailed comments on any problems or difficulties in the rendering and the way in which they were resolved [13]. It was then validated by a bilingual team comprising geriatricians, neurologists, and methodologists competent in the area of study. The APQ is a multi-dimensional assessment tool with 7 dimensions, each comprising 3 to 5 items. The original dimensions, as described by McGee's team, assessing Irish community residents aged 65 and older, were preserved. These dimensions are as follows:

*Timeline*: issues relating to an individual’s awareness of aging and experience of the process over time, with two sub-dimensions: *timeline chronic* (awareness that one’s age or aging is chronic in nature) and *cyclical* (one experiences variation in awareness of aging).

*Consequences*: beliefs about the impact of aging on one’s life across a variety of domains. This has two sub-dimensions: *consequence positive and negative*.

*Control*: beliefs about personal way of managing one‘s experience of aging, with two sub-dimensions: *positive and negative control*.

*Emotional representations*: emotional response generated by aging.

These dimensions form a self-evaluation scale of 32 items scored on a 5-point Likert scale ranging from "strongly disagree" to "strongly agree" (Additional file 1). The dimension scores retained correspond to the mean of responses ranging from 1 to 5. For the negative control dimension only, the response scale is reversed (1 = "strongly agree" and 5 = "strongly disagree"). On subscales with 5 and 4 items, if more than 2 items were missing then a subscale score was not calculated for that variable. For the 3-item subscales, if more than 1 item was missing then a subscale score was not calculated for that variable. Higher scores are indicative of greater endorsement of a specific perception. Respondents for whom half the responses were missing were excluded from the analysis.

## Statistical analysis

### Validation study of the APQ

A first analysis of responses to the questionnaire was performed for those aged 65 or over, in line with the population in which the APQ was validated [10]. A complementary analysis was conducted on the group of respondents aged 55 to 64.

The rate of non-response was calculated for each item and for each respondent so as to detect questions that could be not well understood. A percentage of missing data under 5% was considered acceptable. The study of item distribution looked for items that were not sufficiently informative (floor or ceiling effects). The main purpose of the analysis was to provide support for the factorial structure of APQ questionnaire in a French population using confirmatory factor analysis (CFA). The CFA model was designed to evaluate how the factorial structure of APQ previously defined in the Irish study fitted to the French population. The normality-adjusted chi² test for exact fit was used as the measure of model fit. Because of its overly sensitiveness to sample size, model fit assessment relied on effect size measured by root mean square error of approximation (RMSEA) and by the comparative fit index (CFI). Cut-off values for an acceptable model fit were RMSEA below .08 and CFI above .95 [14]. Modification indices were used as indicators to explore the sources of lack-of-fit. A multi-group confirmatory factor analysis (MGCFA) was performed to test for invariance of the factorial structure between age groups. A likelihood-ratio chi² test was used to compare nested models with or without constraints on identical factor pattern coefficients, designed to assess different levels of measurement invariance [15,16]. Differences between CFI indexes were used to detect lack of metric invariance, based on a cut-off value of .01 for delta-CFI [17].

The dimension scores were calculated according to Barker et *al.* in 2007 [10]. Descriptive statistics, means and standard deviations, were calculated for each of the dimensions. Cronbach's alpha coefficient was calculated to assess internal consistency. The relationships between socio-demographic variables (age, gender, type of education) and the dimensions in the questionnaire were tested at P < .05 significance level. The statistical analyses were performed on SAS 9.2 for Windows and Lisrel 8.80.

## Results

Six hundred and seventy-four respondents agreed to participate in this first OPREVI survey. The participation rate, relative to the population able to participate, was 47%. This rate was similar among people aged <65 (45.7%) and people aged ≥65 (48.5%). Eighteen subjects responded to fewer than 16 items in the APQ and were excluded from the analysis. In all, 656 questionnaires were analysed, corresponding to 286 men and 370 women, of whom 262 were under 65 and 394 were 65 or over. The socio-demographic data are described in Table 1.

**Table 1:** Socio-demographic characteristics of respondents in the study (N = 656)

	**Age <65 yrs (n = 262)**		**Age ≥ 65 yrs (n = 394)**	
	**n**	**%**	**n**	**%**
Gender (N = 656)				
Male	122	46.6	164	41.6
Female	140	53.4	230	58.4
Marital status (N = 656)				
Married or with partner	206	78.6	236	59.9
Separated or divorced	28	10.7	26	6.6
Widowed	19	7.3	118	30.0
Single	9	3.4	14	3.5
Level of education (N = 651)				
No qualifications	7	2.7	63	16.2
Primary school certificate	51	19.5	143	36.7
CAP, BEP (vocational training)	93	35.6	72	18.5
Brevet des collèges, BEPC (lower secondary)	26	10.0	36	9.2
Baccalauréat (general)	17	6.5	26	6.7
Baccalauréat technical or vocational	19	7.3	10	2.6
Higher education diploma	42	16.1	30	7.7
Other	6	2.3	10	2.6
Age (yrs) (N = 656) Mean (sd) [min-max]	59.8 (2.8) [54–64]		77.4 (8.2) [65–106]	

There was no gender disparity among respondents and non-respondents. There was a mean age difference, as the non respondents were younger (69.1 ± 10.0 *vs.* 70.7 ± 10.9 yrs; P < .005). We think that the third party that could assist the elderly to answer the questions, allowed this rate of participation in the older.

### The APQ structure among respondents aged 65 and over

The validation of the questionnaire was performed on 164 men and 230 women aged 65 and over. The response rate was over 97% for all items (amounting to between 2 and 12 missing answers to items). Three hundred and forty eight subjects (88.3%) responded to all the items. Responses to items in the questionnaire were evenly spread across the response scale with no evidence of saturation.

**Figure 1:**
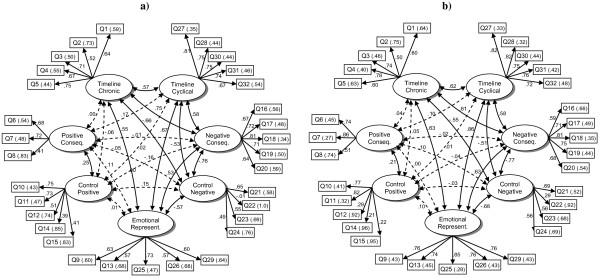
**APQ questionnaire confirmatory factor analysis in subjects 65 or older (Figure**1**a) and in subjects aged 55 to 65 (Figure**1**b).** On this figure, ovals represent factors and rectangles questionnaire items. Inside rectangles are given the item number (see Additional file 1 for litteral items) and its standardized error variance estimate in parentheses. Unidirectional arrows represent associations between factors and items according to the CFA model with standardized factor loadings estimates. Bidirectional arrows represent factor correlations with their estimates; dotted lines are associated with low correlations (< .20). *Conseq. Positive and Conseq. Negative: sub-dimensions consequences positive and consequence negative Emotional represent.: dimension emotional representations.*

The CFA model with seven factors failed to perform exact fit to the observed data according to the chi² test, (χ² = 1204.6 df = 443 P < .001). An acceptable fit was indicated by RMSEA = .070 whereas the CFI value (.93) was slightly below the suggested cut-off. The factor loadings (Figure 1a) were coherent with original findings with an exception concerning item 22 which did not get loading from the *control negative* factor. Nevertheless removing this item had no impact on global model fit, thus suggesting that improvement should be expected from a better translation of the original item. Exploring modification indices suggested that an improvement in model fit could be obtained by introducing non-unique factor loadings on several items and by adding inter-item covariance parameters. These a-posteriori suggested modifications improved model fit based on the chi² value, but failed to make sense theoretically; none of them clearly improved RMSEA and CFI fit indices. The higher mean dimension score was 3.87 ± 0.72 for *control positive* and the lower 2.45 ± 0.94 for *emotional representations* (Table 2). Values were all markedly spread across the scale as a whole without saturation. Internal consistency, as evaluated by the values of the Cronbach alpha coefficient, ranged from .52 to .86.

**Table 2:** Descriptive statistics of the Aging Perception Questionnaire dimensions among respondents aged 65 or over (N = 394) and under 65 (N = 262)

	***Timeline chronic***	***Timeline cyclical***	***Consequences positive***	***Consequences negative***	***Emotional representations***	***Control positive***	***Control negative***
**Results McGee 2007 (age ≥65 yrs, n = 2033)**							
Mean	2.90	2.70	3.70	3.40	2.40	3.80	2.65
Sd	0.87	0.82	0.59	0.74	0.74	0.54	0.71
**Results this study 2011 (age ≥65 yrs, n = 394)**							
N	391	389	389	393	391	391	392
Mean	3.02	2.88	3.22	3.46	2.45	3.87	3.12
Sd	0.97	1.09	0.81	1.04	0.94	0.72	0.74
α	0.80	0.86	0.63	0.83	0.76	0.70	0.52
**Results this study 2011 (age <65 yrs, n = 262)**							
N	262	262*	262	262	262	262	262
Mean	2.35	2.47	3.37	2.44	2.31	4.10	3.51
Sd	0.82	1.01	0.84	0.93	0.97	0.55	0.70
α	0.77	0.88	0.75	0.82	0.88	0.62	0.59
p (age ≥65 yrs vs. age <65 yrs)	<0.0001	<0.0001	0.018	<0.0001	0.095	<0.0001	<0.0001

### The APQ structure among respondents aged 55 to 64

This group involved 122 men and 140 women aged 55 to 64. The response rate was over 98% for all items (amounting to between 0 and 4 missing answers to items at most). Two hundred and forty subjects (91.6%) completed the full questionnaire. The responses are spread across the scale with no saturation effect.

The same seven-factor a-priori-defined model yielded a similar, but slightly better fit in the 55–64 as in the older group (RMSEA = .069; CFI = .95), but also failed to perform exact fit (χ² = 953.3 df = 443 P < .001). Factor loadings were mostly similar in the two age groups except in the two control dimensions (Figure 1b). The three last items in *control positive* had lower, despite significant loading standardized estimates whereas the second item in *control negative* had a larger loading. Cronbach alpha coefficient ranged between .59 for *control negative* and .88 for *timeline cyclical* and *emotional representations* (Table 2).

### Factorial invariance between age groups

A first seven-factor MGCFA model assuming different parameters in the two age groups was applied with χ² = 2179.9 (df = 904 P < .001) and with indices of approximate fit RMSEA = .069 and CFI = .94. A second MGCFA model constraining the factor loadings to be the same in the two age groups resulted in a significantly larger χ² value (χ² = 2288.1 df = 929 P < .001; Δχ² = 108.3 df = 25 P < .001) with a moderately larger RMSEA = .071 and the same CFI = .94 (ΔCFI = .00).

### Influence of age and gender on mean scores

Perceptions of aging evolved with age. Significant correlations with age were observed among those aged 65 and over for every dimension, except *consequences positive*. Conversely, in younger participants, no dimension correlated with age (Table 3). The scores relating to *consequences negative* were significantly higher among subjects of 65 and over (3.46 *vs* 2.44, p < .0001) as were those for the dimension *timeline chronic* (3.02 *vs* 2.35, p < .0001) (Table 2). The scores for the *consequences positive* dimension differed slightly between respondents aged 65 and over and those under 65 (3.22 *vs* 3.37, p = .018). The scores for the dimension *control negative*, where the scoring system is the reverse of the other dimensions, is significantly lower among subjects aged 65 and over (3.12 *vs* 3.51, p < .0001).

**Table 3:** Aging Perception Questionnaire dimensions and socio-demographic characteristics: univariate analysis of scores

	**Timeline chronic**	**Timeline cyclical**	**Consequences positive**	**Consequences negative**	**Emotional representations**	**Control positive**	**Control negative**
Age: 65 and older							
Age							
Coefficient of correlation	0.30	0.28	−0.07	0.55	0.13	−0.18	−0.26
p	<0.0001	<0.0001	0.15	<0.0001	0.0093	0.0003	<0.0001
Sex							
Female (n = 230)	2.96	3.02	3.22	3.53	2.44	3.83	3.08
Male (n = 164)	3.11	2.68	3.21	3.35	2.45	3.93	3.18
p	0.11	0.0026	0.97	0.091	0.93	0.18	0.22
Marital status							
Married (n = 236)	2.97	2.80	3.24	3.33	2.40	3.89	3.18
Single (n = 158)	3.10	3.00	3.18	3.66	2.51	3.85	3.04
p	0.19	0.77	0.53	0.0019	0.29	0.63	0.053
Level of education							
< Baccalauréat (314)	3.08	2.91	3.20	3.51	2.48	3.84	3.08
> = Baccalauréat (n = 66)	2.71	2.73	3.29	3.22	2.30	4.01	3.31
p	0.0050	0.22	0.37	0.039	0.18	0.081	0.023
Age: 55-64							
Age							
Coefficient of correlation	0.03	−0.07	0.00	0.01	−0.05	−0.02	−0.07
p	0.61	0.28	0.99	0.87	0.43	0.80	0.28
Sex							
Female (n = 140)	2.27	2.59	3.44	2.45	2.48	4.18	3.48
Male (n = 122)	2.45	2.33	3.30	2.43	2.13	4.01	3.55
p	0.084	0.038	0.18	0.84	0.0033	0.013	0.41
Marital status							
Married (n = 206)	2.33	2.47	3.36	2.43	2.31	4.10	3.50
Single (n = 56)	2.42	2.46	3.44	2.46	2.35	4.11	3.56
p	0.46	0.92	0.50	0.84	0.79	0.82	0.52
Level of education							
< Baccalauréat (n = 177)	2.39	2.42	3.35	2.51	2.29	4.05	3.45
> = Baccalauréat (n = 78)	2.30	2.55	3.47	2.30	2.35	4.22	3.65
p	0.41	0.32	0.29	0.11	0.65	0.020	0.041

Significant differences occurred between men and women of 65 and over and under 65 for *timeline cyclical*, (2.68 vs. 3.02, p = .0026 and 2.33 vs. 2.59 p = .0026) with higher scores among women (Table 3).

## Discussion

The validation of the French version of the APQ was conducted in a population aged 65 and over, and its psychometric properties were then studied in a younger group of respondents aged 55 to 64. The results of CFA provided additional support for the multidimensional structure of the perception of aging, with 7 factors that were superimposable on those described by the original team from an Irish population. The investigation of the psychometric properties of the French version also showed good internal consistency across these dimensions.

The factorial model estimated using CFA did not perfectly fit the observed data as shown by the highly significant chi² test for exact fit, but this test is known to be overly sensitive to sample size. Approximate fit indices have been proposed to overcome this difficulty. The value of CFI = .93 in people age 65 and over did not pass the standard cut-off of .95 [14] whereas CFI = .95 in those age 55–64 equated this limit. Item 22 in the *control negative* scale did not perform well; that was interpreted as a need for an improved translation of this item.

MCFA allowed testing for invariance between the two age groups. The chi² test comparing nested factorial models rejected measurement invariance in relation with differences in some parameters of the model. However the comparison of model fit with ΔCFI = .00 compared to a cut-off value of .02 was a good evidence in favor of approximate factorial invariance between age groups. This result is important in the view of extending the use of the APQ questionnaire in younger people, but further evidence is needed from new independent data.

The participation rate of 47% could be considered low. Non-responders answers might vary as to the distribution of perceptions of ageing compared to responders. Although not strictly representative, our sample was nevertheless close to being representative of the French general population (http://www.insee.fr). When we took the 3 major determinants (age, gender, level of education) into account, there was no gender difference between responders and non-responders and the percentage of males in our sample was close to that for the French population in both age groups: 47% of responders (52% in France overall) for subjects < 65 and 42% of responders (45% in France overall) for subjects aged ≥65. The proportion of subjects who were single was 21% among participants aged < 65 and 40% among responders aged ≥ 65, compared to 20% and 49% respectively in the French general population. The proportion of participants with a high level of education (at least the baccalaureat level) was 32% among responders < 65 (29% in France) and 19% among participants ≥ 65 (the same as in France).

The APQ multidimensional scale provides a new approach for the understanding of the perception of aging. Its relatively stable factorial structure gives a basis to explore the way it is varying or not in the seven dimensions among people of different ages.

The perception of the *negative consequences* of aging evolves with the age of respondents. The older the respondent, the greater the awareness of these negative consequences of growing old, and the risk of decline [18]. There is a progressive realisation of the risk of dependency and loss of the ability for action over time, and more generally if the losses and renunciations are linked to aging. This negative view of aging may be specific to western societies [19].

The way the *positive consequences* of aging are perceived appears identical whatever the age of the respondents. All respondents subscribed to certain advantages in growing old - time for thought, wisdom, making new acquaintances, leisure [19,20]. Despite lesser scope for action, this does not prevent greater wisdom and appreciation of life. Personal identity is emphasised in the *positive consequences*, while the *negative consequences* tend to emphasise the societal weight of aging. Thus these two dimensions do not mirror one another, but reflect two different perceptions which will lead to corresponding interventional strategies. Thus the respondents getting older have a realistic view of the decrease in their ability for action and of the need to relinquish certain things as one grows old. They believe in the preservation of their intrinsic qualities. There emerges an anthropology of aging in which societal influences confirming the loss of the ability for action are mingled with the personal desire to find intrinsic qualities in growing old.

The timeline and control dimensions were also significantly different between people ≥65 and the younger. In the face of growing old, the individual adapts in order to accept. The awareness of aging is more often constant than cyclical in the oldest (3.02 and 2.88), while this awareness goes more often through phases of feeling old in the youngest (timeline chronic 2.35, timeline cyclical 2.47). The highest dimension score occurs in the control-positive dimension, no matter the age, showing that all respondents believe they have a control over the positive experience of aging. Respondents <65 had also a high score in the control-negative dimension (3.51) showing that they also assume control over negative aspects of aging.

The *Control positive* dimension is clearly individualised among the older as in the youngest respondents. However, positive control in the younger respondents shows that they do not subscribe to a degree of autonomy with regard to society, and do not yet envisage the need for others. [18,21]. *Control negative* appears more important in the older group as they have to cope every day with their functional decline.

Aging is a dynamic process that is grounded in individual and societal references. The fear of dependency and the perception of one's personal identity within society on the part of older individuals make it possible to envisage medico-social interventions. Those who have a favorable perception of growing old are probably able to make the right decisions at the right moment. Thus, to combat the fear of becoming dependent, those with a pessimistic outlook towards aging could be stimulated in their ability for action and encouraged to open up to others.

## Conclusion

This study, being conducted among French participants aged 65 and over, confirmed that the French translation of the APQ has a multidimensional structure, which is superimposed to the dimensions of the original Irish version. The same factorial structure applies acceptably to the younger group (aged 55–64). The OPREVI study is ongoing, and will collect data on the physical, material and social characteristics of participants. It will therefore be possible to analyse the variables associated with the perceptions of aging. On the basis of an individual's perceptions of aging as captured by this questionnaire, and his or her clinical profile, tailored multi-dimensional assistance could be made available aiming to provide incentive to anticipate or to adapt to difficulties.

## Competing interests

The authors declare that they have no competing interest.

## Authors’ contributions

II, JLH, RG, PI and MP substantially contributed to the conception, design, analysis and interpretation of data and have been involved in revising the manuscript critically for important intellectual content and have given final approval of the version to be published. HMG reviewed the manuscript and has given final approval of the version to be published. All authors read and approved the final manuscript.

## Pre-publication history

The pre-publication history for this paper can be accessed here:

http://www.biomedcentral.com/1471-2318/12/17/prepub

## Supplementary Material

Additional file 1 **Aging Perception Questionnaire perception of aging scale (English original measure [**[10]**] and the French translation).** Click here for file
