# Supplementary material for: The validation of a French-language version of the Aging Perceptions Questionnaire (APQ) and its extension to a population aged 55 and over
Source: BMC Geriatr. 2012 Apr 30;12:17. doi: 10.1186/1471-2318-12-17 (PMC3459801; doi:10.1186/1471-2318-12-17)
Supplement: Additional file 1 — Aging Perception Questionnaire perception of aging scale (English original measure [[10]] and the French translation). [file 1471-2318-12-17-S1.rtf]

Additional file: Aging Perception Questionnaire perception of aging scale (English original measure [10] and the French translation)

Timeline (chronic)	
I am conscious of getting older all of the time	1-Je me rends compte que je vieillis un peu plus tous les jours	
I am always aware of my age	2-Je suis tout le temps conscient(e) de mon âge	
I always classify myself as old	3-Je me considère toujours comme étant vieux(vieille)	
I am always aware of the fact that I am getting older	4-J'ai toujours en tête le fait que je vieillis	
I feel my age in everything that I do	5-Je ressens les effets de mon âge dans tout ce que je fais	
Consequences positive	
As I get older I get wiser	6- En vieillissant je gagne en sagesse	
As I get older I continue to grow as a person	7- J'ai l'impression de me bonifier avec l'âge	
As I get older I appreciate things more	8- En vieillissant, je sais mieux apprécier les choses	
Control positive	
The quality of my social life in later years depends on me	10- Les contacts que je pourrai avoir avec les autres, au fur et à mesure que je vieillis dépendent d'abord de moi	
The quality of my relationships with others in later life depends on me	11- La qualité de mes relations avec les autres au fur et à mesure que je vieillis dépend d'abord de moi	
Whether I continue living life to the full depends on me	12- Il ne dépend que de moi de profiter au maximum de la vie	
As I get older there is much I can do to maintain my independence 	14- Avec l'âge, il y a beaucoup de choses que je peux faire pour conserver mon indépendance	
Whether getting older has positive sides to it depends on me	15- C'est à moi de faire en sorte qu'avec l'âge la vie garde des côtés positifs	
Consequences negative	
Getting older restricts the things that I can do	16- En prenant de l'âge, il y a moins de choses que je suis encore capable de faire	
Getting older makes me less independent	17- En prenant de l'âge, j'ai moins d'indépendance	
Getting older makes everything a lot harder for me	18- Avec l'âge, tout devient beaucoup plus difficile	
As I get older I can take part in fewer activities	19- Avec l'âge, les activités auxquelles je peux participer sont de moins en moins nombreuses	
As I get older I do not cope as well with problems that arise	20- En vieillissant, je gère moins bien les problèmes	
Control negative	
Slowing down with age is not something I can control	21- Le ralentissement dû à l'âge est quelque chose que je ne peux pas contrôler	
How mobile I am in later life is not up to me	22- Avec l'âge, les possibilités pour me déplacer à la maison et à l'extérieur ne dépendent pas de moi	
I have no control over whether I lose vitality or zest for life as I age	23- Si en vieillissant, je perds ma vitalité ou mon goût de vivre, je n'y peux rien	
I have no control over the effects which getting older has on my social life	24- Je ne peux pas contrôler les conséquences du vieillissement sur ma vie avec les autres	
Emotional representations	
I get depressed when I think about how aging might affect the things that I can do	9- Cela me déprime de penser qu'avec l'âge il y a des choses que je ne pourrai plus faire	
I get depressed when I think about the effect that getting older might have on my social life	13- Cela me déprime de penser aux conséquences que mon âge pourrait avoir sur mes possibilités de contact avec les autres	
I get depressed when I think about getting older	25- Cela me déprime de penser au vieillissement	
I worry about the effects that getting older may have on my relationships with others	26- Les conséquences que pourrait avoir mon âge sur mes relations avec les autres me tracassent	
I feel angry when I think about getting older	29- Cela me révolte de penser que je vieillis	
Timeline (cyclical)	
I go through cycles in which my experience of aging gets better and worse	27- Il y a des périodes où je vis bien le fait de vieillir, et des périodes où je le vis mal	
My awareness of getting older comes and goes in cycles	28- Le sentiment que j'ai de vieillir va et vient de manière périodique	
I go through phases of feeling old	30- Il y a des moments où je me sens vieux/vieille, et puis cela passe	
My awareness of getting older changes a great deal from day to day	31- La conscience que j'ai de prendre de l'âge varie beaucoup d'un jour à l'autre	
I go through phases of viewing myself as being old	32- Il y a des périodes où je me considère comme étant vieux/vieille	
